# Racial Differences in Self-Report of Mental Illness and Mental Illness Treatment in the Community: An Analysis of Jail Intake Data

**DOI:** 10.1007/s10488-023-01297-4

**Published:** 2023-09-21

**Authors:** Narcissa Plummer, Rubeen Guardado, Yvane Ngassa, Cristina Montalvo, Peter J. Kotoujian, Kashif Siddiqi, Thomas Senst, Kevin Simon, Andrea Acevedo, Alysse G. Wurcel

**Affiliations:** 1 Department of Population Health, Northeastern University, Boston, MA USA; 2 Division of Geographic Medicine and Infectious Diseases, Department of Medicine, Tufts Medical Center, Boston, MA USA; 3 Department of Psychiatry, Tufts Medical Center, Boston, MA USA; 4 Middlesex Sheriff’s Office, Billerica, MA USA; 5 Harvard Medical School, Boston, MA USA; 6 Children’s Hospital, Boston, MA USA; 7 Department of Community Health, Tufts University, Medford, MA USA; 8 Tufts University School of Medicine, Boston, MA USA

**Keywords:** Race, Ethnicity, Incarcerated, Jail, Prison, Health disparity, Mental illness

## Abstract

Jails and prisons in the United States house people with elevated rates of mental health and substance use disorders. The goal of this cross-sectional study was to evaluate the frequency of racial/ethnic differences in the self-report of mental illness and psychiatric medication use at jail entry. Our sample included individuals who had been incarcerated between 2016 and 2020 at the Middlesex Jail & House of Correction, located in Billerica, MA. We used data from the “Offender Management System,” the administrative database used by the jail containing data on people who are incarcerated, and COREMR, the electronic medical record (EMR) used in the Middlesex Jail & House of Correction. We evaluated two primary outcomes (1) self-reported mental illness history and (2) self-reported use of psychiatric medication, with the primary indicator of interest as race/ethnicity. At intake, over half (57%) of the sample self-reported history of mental illness and 20% reported the use of psychiatric medications. Among people who self-reported a history of mental illness, Hispanic (AOR: 0.73, 95% CI: 0.60–0.90), Black (AOR: 0.52, 95% CI: 0.43–0.64), Asian/Pacific Islander (Non-Hispanic) people (AOR: 0.31, 95% CI: 0.13–0.74), and people from other racial/ethnic groups (AOR: 0.33, 95% CI: 0.11–0.93) all had decreased odds of reporting psychiatric medications. Mental illness was reported in about one-half of people who entered jail, but only 20% reported receiving medications in the community prior to incarceration. Our findings build on the existing literature on jail-based mental illness and show racial disparities in self-report of psychiatric medications in people who self-reported mental illness. The timing, frequency, and equity of mental health services in both the community and the jail setting deserves further research, investment, and improvement.

**Supplementary Information:**

The online version contains supplementary material available at 10.1007/s10488-023-01297-4.

## Introduction

The number of people with mental illness who have contact with the criminal-legal system is increasing. (Baranyi et al., 2022; Butler et al., 2022) Rates of mental illness and substance use disorder in jails are several times that of people living in the community with no history of incarceration. (Baranyi et al., 2022; Fazel & Danesh, 2002; Gottfried & Christopher, 2017; Rich et al., 2011; Yi et al., 2017). About 12 million people go through a jail or prison annually (the majority of whom enter the system through local jails). Of those people, one-third who are identified with a mental illness have been diagnosed before and during incarceration. (Kaba et al., 2015) In June 2017, the Bureau of Justice Statistics reported from 2011–2012, 26% of jail inmates met the threshold for serious psychological distress in comparison to 5% of the general population. (Bronson & Berzofsky, 2017) The Bureau of Justice Statistics also estimated from 2007–2009, 63% of people who had been sentenced to jail had drug use disorders compared to 5% of the general, adult population. (Bronson et al., 2017) There is newer data available for prisons—showing 43% of people who are incarcerated in state prisons had a history of mental illness with 53% of White people and 33% of Black people reporting mental illness. (Assistance TBoJ, 2021)

Treatment of mental illness and substance use disorder are associated with better quality of life (Golan et al., 2022), increased time in recovery (Sigmon et al., 2016; Winetsky et al., 2020), decreased re-incarceration rates (Evans et al., 2022), and decreased mortality (Springer et al., 2018). As a method to better track the support provided to people with mental illness in the criminal-legal system, the sequential intercept model (SIM) was developed. This model specifically provides a framework of contacts or “intercepts” for people with mental illness in the criminal-legal system including: community (Intercept 0), law enforcement (Intercept 1), court hearings and jails (Intercept 2 & 3), re-entry (Intercept 4) (Munetz & Griffin, 2006), and community supervision (e.g., parole and probation) (Intercept 5) (Associates PR, 2023). For example, interventions to improve linkage to mental health treatment in the community have proven to be a successful strategy to prevent re-incarceration (Smith et al., 2022; Stewart et al., 2022). However, one aspect of intervening at “Intercept 2” is that the identification and treatment of mental illness (Administration SAaMHS, 2019), including substance use disorder, has been challenging to evaluate thus far (Martin et al., 2018a; Simpson & Jones, 2018). Two of the challenges most prevalent are the usage of multiple mental health screening tools (Martin et al., 2018a, 2018b; Simpson & Jones, 2018) and structural barriers (knowledge of screening tools, access to screening tools, and cost) (Martin et al., 2018a).

Mental illness disparately impacts vulnerable populations in larger society, including but not limited to, racially and ethnically minoritized populations (including but not limited to Black, Hispanic, Asian, and Native American people), lesbian, gay, bisexual, transgender, and queer (LGBTQ+) populations, people living in poverty, and individuals with criminal-legal involvement (Alvidrez & Barksdale, 2022; Carda-Auten et al., 2022; Chow et al., 2003; General OotS & Services CfMH, 2001; Goldbach et al., 2022; Hedden et al., 2021; Prokosch et al., 2022; Youman et al., 2010). There is considerable intersectionality between these groups of people, leading to an increased risk of mental health disparity within these vulnerable populations. For example, institutionalized racism perpetuates the overrepresentation of Black and Latinx populations among impoverished, unhoused, and low socioeconomic status groups, rendering them less likely to receive timely and appropriate mental health services (Alvidrez & Barksdale, 2022; Hoptman et al., 1999; Paradies et al., 2015; Saldana et al., 2021). Racial disparities continue to affect the accuracy of mental health diagnosis and treatment even after revisions on the diagnostic criteria for psychiatric disorders (bipolar disorder, major depression, and schizophrenia) (Akinhanmi et al., 2018; Saldana et al., 2021).

There is less data about access to if and how race and ethnicity impacts mental health assessment in jails in comparison to the public. The mistreatment by the health care system has created mistrust within racially and ethnically minoritized populations. Some people from racially and ethnically minoritized populations experiencing mental illnesses are now more likely to seek aid from family and religious providers instead of formal mental health services (Sheehan et al., 2018). In a cohort of 6673 people diagnosed with mental illness during incarceration, 39% of Black people were placed in solitary confinement compared to 9% of White people (see Kaba et al., 2015). White people in the community were more likely to report pre-incarceration mental health treatment compared to Black people, although no difference was found by race in utilizing and accessing mental-health treatment in jail (see Kaba et al., 2015; Youman et al., 2010). A more recent study (see Hedden et al., 2021) of over 600 people with mental illnesses in Midwestern jails reported no racial differences in jail-based mental health treatment, but White race was associated with a 1.9 times increased odds of connecting with post-incarceration mental health care (Hedden et al., 2021).

The first medical interaction to occur when individuals with criminal-legal involvement arrive to jail is the intake process. Individuals are asked questions about their mental health history, active mental health issues, and all medications (Carda-Auten et al., 2022). To date, there is little data about the intake process in jails and how individuals with criminal-legal involvement report their past, or current mental illnesses as a reflection of their access to mental health care in the community. The goal of this study is to evaluate the frequency of self-report of mental illnesses at jail entry and evaluate racial differences in self-report of mental illnesses and mental illness treatments prior to incarceration, in a cross-sectional study of people entering a county facility in Massachusetts.

## Materials and Methods

### Study Site and Sample

The Middlesex Jail & House of Correction**,** located in Billerica, MA, has an average daily capacity of 1150 people and houses both pre-trial (detained) and incarcerated (sentenced) people. There were about 22,000 incarcerations in the study period (2016–2020). The jail holds roughly 80% pre-trial (detained) and 20% incarcerated (sentenced) people. The racial breakdown of the jail is approximately 50% White, 25% Black, and 25% Hispanic/Latinx (Middlesex County Jail, 2022). There are no major differences in pre-trial vs. sentenced by race or ethnicity. Our sample included individuals who had an incarceration that occurred between 2016 and 2020. Although individuals could have multiple incarcerations during this period, our analytic sample was made up of individuals (one record per person).

### Current Mental Health Screening

The Middlesex Jail & House of Correction has a Mental Health Director and six, full-time licensed clinicians and three per-diem clinicians. Mental Health Services are available seven-days per week. Monday through Friday, 7 a.m. to 10 p.m, and eight-hours on Saturday and Sunday. When an individual in custody enters the facility, they are screened by a medical staff member within the first hour (via the nursing intake) for mental health needs. Each day, a mental health clinician reviews medical screening done the night before and each person is offered a comprehensive Mental Health Evaluation and Treatment Plan completed by a licensed mental health staff member, LICSW or LMHC. Mental health services offered include: individual therapy (Cognitive Behavioral or Solution Focused and Trauma Centered), group therapy, crisis intervention, clinical psychopharmacology, and referrals to the Department of Mental Health or Department of Developmental Services, Community Mental Health Partners through the Mass Behavioral Health Justice Initiative (BHJI).

### Data Source

With the assistance of Informational Technology (IT) specialists at the Middlesex Sheriff’s Office and the medical record management company, we merged and de-identified two datasets at the Middlesex Sheriff’s Office: (1) “Offender Management System,” the administrative database used by the jail to store data on those who are incarcerated and (2) CorEMR, the electronic medical record (EMR) used at the Middlesex Jail & House of Correction. Data drawn from the administrative database included: demographics, (race/ethnicity, age, country of birth), date of incarceration, and date of release. Data obtained from CorEMR included data from the medical intake questionnaire and medications reported at intake. The intake questionnaire includes elements related to travel history, history of incarceration, alcohol consumption and drug usage, (history, detox, seizures, blackouts), mental health screening, (diagnoses and treatment history), health insurance, medical history, release information, TB screening, medications, allergies, testing, (Hep C and HIV), and vaccination. Exclusion criteria for the cohort included unclassified, or missing race data, missing country of birth, and missing self-reported history of psychiatric illness.

### Variables

Our analysis evaluated two primary outcomes (1) self-reported mental illness history and (2) self-reported psychiatric medication, both assessed during intake. Self-reported mental illness was determined using the answer to the nursing intake form question, “Do you have a history of mental illness?” As we defined each intake experience as an opportunity to identify mental illness, we use the incarceration intake as the unit of analysis. If a person was incarcerated multiple times during the study period, we classified an answer of “yes” to this question on any of the intake forms as having a history of mental illness. Report of medications for mental illness was determined based on review of a pharmacy-verified medication list which was entered as free text during nursing intake. The list was reviewed by a clinician (AGW) who determined if the medication was related to mental health (Y/N). If someone was incarcerated several times, then report of mental health medications during one incarceration period qualified for this outcome.

The primary indicator of interest was race/ethnicity (categorical). Race/ethnicity was entered at intake as: White, Black, Hispanic (no race indicated), Asian/Pacific Islander (Non-Hispanic), American Indian, Other, and Unknown. We categorized race/ethnicity as White (Non-Hispanic), Black (Non-Hispanic), Hispanic, Asian/Pacific Islander (Non-Hispanic) and “Other” race/ethnicity. We excluded those categorized as Unknown race as there was not a clear identifier for race/ethnicity. Covariates included were age and country of birth (dichotomized as foreign-born vs. U.S.-born). Age was categorized into three groups: 18–29 years old, 30–44 years old, and ≥ 45 years old.

### Statistical Analyses

We first conducted descriptive statistics of the sample. Next, we conducted cross-tabulations of our outcome variables with race/ethnicity and each of our predictor variables. We then conducted a simple logistic regression analysis followed by a multivariable logistic regression analysis predicting each outcome. Our multivariable logistic regression models included our main independent variable (race/ethnicity) and our covariates (age and the foreign-born indicator variable). In the analyses predicting history of psychiatric medications, we only included those who had reported a history of mental illness at intake, although we also conducted a sensitivity analysis using the entire sample. The analysis was performed using Stata/SE 17.0.

## Results

The final analytic sample included 7884 individuals with criminal-legal involvement, excluding people who were unclassified, or missing race data (n = 171), missing country of birth (n = 39), and missing self-identified history of psychiatric illness (n = 378). The sample was 58% White (Non-Hispanic), 18% Black (Non-Hispanic), and 21% Hispanic. The mean age of the sample was 39-years old (SD = 11.5). Over half (57%) of the sample self-reported history of mental illness at intake and 20% reported psychiatric medications at intake (Table 1). Additionally, almost half (47%) of the sample were incarcerated more than once, and collectively they had 15,403 incarcerations with an average length of incarceration of 90 days (results not shown).

**Table 1:** Demographic Characteristics (2016–2020), N = 7884

Variable	N (%)
*Age*	
18–29 years old	1881 (24)
30–44 years old	3623 (46)
≥ 45 years old	2380 (30)
*Race*	
White, Non-Hispanic	4631 (58)
Black, Non-Hispanic	1385 (18)
Hispanic	1661 (21)
Asian/Pacific Islander (Non-Hispanic)	131 (2)
Other race/ethnicity	76 (1)
*Region of Birth*	
North America	6602 (83)
Latin American & Caribbean	987 (12)
Europe & Central Asia	102 (1)
Sub-Saharan Africa	97 (1)
East Asia & Pacific	66 (< 1)
Middle East & North Africa	19 (< 1)
South Asia	11 (< 1)
*Foreign-born*^a^	1282 (16)
*Ever self-reported mental illness*	4542 (57)
*Ever self-reported psychiatric medications on intake*	1552 (20)

^a^Outside of the 50 U.S. States and Puerto Rico

In the multivariable analysis, Hispanic (AOR: 0.75, 95% CI: 0.66–0.86), Black (Non-Hispanic) (AOR: 0.56, 95% CI: 0.50–0.64), and Asian/Pacific Islander (Non-Hispanic) individuals (AOR: 0.47, 95% CI: 0.32–0.68) had decreased odds of self-reporting mental illness on intake compared to White (Non-Hispanic) individuals (Table 2). Among individuals who self-reported a history of mental illness, Hispanic (AOR: 0.73; 95% CI: 0.60–0.90), Black (AOR: 0.52, 95% CI: 0.43–0.64), Asian/Pacific Islander (Non-Hispanic) individuals (AOR: 0.31, 95% CI: 0.13–0.74), and individuals from other racial/ethnic groups (AOR: 0.33, 95% CI: 0.11–0.93) all had decreased odds of reporting psychiatric medications compared to their White counterparts (Table 3).

**Table 2:** Bivariate and Multivariable Regression Analysis of Those Who Self-Reported Mental Health Illness on Intake by Sociodemographic Characteristics

	Cross-tabulations		Bivariate regression analyses		Multivariable regression analyses	
	No (N = 3342) N (%)	Yes (N = 4542) N (%)	OR (95% CI)	P-value	AOR (95% CI)	P-value
*Race*						
White, Non-Hispanic	1640 (35)	2991 (65)	Reference		Reference	
Black, Non-Hispanic	718 (51)	667 (48)	0.51 (0.45–0.58)	< 0.001	0.56 (0.50–0.64)	< 0.001
Hispanic	861 (51)	800 (48)	0.51 (0.46–0.57)	< 0.001	0.75 (0.66–0.86)	< 0.001
Asian/Pacific Islander (Non-Hispanic)	82 (62)	49 (37)	0.33 (0.23–0.47)	< 0.001	0.47 (0.32–0.68)	< 0.001
Other race/ethnicity	41 (54)	35 (46)	0.47 (0.30–0.74)	0.001	0.69 (0.43–1.11)	0.124
*Age*						
18–29 years	849 (45)	1032 (55)	Reference		Reference	
30–44 years	1452 (40)	2171 (60)	1.23 (1.10–1.38)	< 0.001	1.19 (1.06–1.34)	0.003
≥ 45 years	1041 (44)	1339 (56)	1.06 (0.94–1.20)	0.362	0.98 (0.86–1.11)	0.720
*Foreign-born*^a^						
No	2534 (38)	4068 (61)	Reference		Reference	
Yes	808 (63)	474 (37)	0.37 (0.32–0.41)	< 0.001	0.43 (0.37–0.50)	< 0.001

^a^Outside of the 50 US States and Puerto Rico; *OR* odds ratio, *AOR* adjusted odds ratio

**Table 3:** Bivariate and Multivariable Analyses of Reported Psychiatric Medications on Intake by Sociodemographic Characteristics Among Self-Reported Mental Health History

	Cross-tabulations		Bivariate regression analyses		Multivariable regression analyses	
	No (N = 3212) N (%)	Yes (N = 1330) N (%)	OR (95% CI)	P-value	AOR (95% CI)	P-value
*Race*						
White, Non-Hispanic	1996 (67)	995 (33)	Reference		Reference	
Black, Non-Hispanic	536 (80)	131 (20)	0.49 (0.40–0.60)	< 0.001	0.52 (0.43–0.64)	< 0.001
Hispanic	606 (76)	194 (24)	0.64 (0.54–0.77)	< 0.001	0.73 (0.60–0.90)	0.008
Asian/Pacific Islander (Non-Hispanic)	43 (88)	6 (12)	0.28 (0.12–0.66)	0.004	0.31 (0.13–0.74)	0.037
Other race/ethnicity	31 (89)	4 (11)	0.26 (0.09–0.74)	0.011	0.33 (0.11–0.93)	0.037
*Age*						
18–29 years old	822 (80)	210 (20)	Reference		Reference	
30–44 years old	1500 (69)	671 (31)	1.75 (1.47–2.09)	< 0.001	1.64 (1.37–1.96)	< 0.001
≥ 45 years old	890 (66)	449 (34)	1.97 (1.63–2.39)	< 0.001	1.82 (1.50–2.21)	< 0.001
*Foreign-born*^a^						
No	2846 (70)	1222 (30)	Reference		Reference	
Yes	366 (77)	108 (23)	0.69 (0.55–0.86)	0.001	0.84 (0.65–1.09)	0.195

^a^Outside of the 50 US States and Puerto Rico; *OR* odds ratio, *AOR* Adjusted odds ratio

We found that 222 individuals report a history of no mental illness, yet reported the use of psychiatric medications. As a sensitivity analysis, we conducted the same analyses predicting psychiatric medications but including the entire sample. Our results were very similar to when we limited our analyses to only those reported a history of mental illness (See Table 1 in the supplemental materials).

## Discussion

Through investigation of a novel dataset, we found that people who are Hispanic, Black (Non-Hispanic), Asian/Pacific Islander (Non-Hispanic), and those from other race/ethnicities were less likely to report a history of mental illness on a jail nursing intake, and less likely to report receiving psychiatric medications for mental health in the community. Mental illness is stigmatized, and self-report of mental illness in illnesses in jails and prisons reflect both the person’s view of their mental health and their view of the facility’s ability to treat their mental illnesses.

Our findings were consistent with the existing literature on jail-based mental illness that have found evidence of racial disparities in mental illness identification in jails (Hedden et al., 2021; Kaba et al., 2015; Martin et al., 2018). As race is a social construct (Flanagin et al., 2021), we do not want to project any oversimplifications of our findings to reflect genetic differences in predisposition to mental illness. There is robust data to support that the experience and manifestation of mental illnesses are influenced by the intergenerational impact of structural racism, cumulative trauma, genetics, epigenetics, and the social and environmental determinants of mental health (Bale & Jovanovic, 2021; Bernardini et al., 2021; Compton & Shim, 2015; Hankerson et al., 2022; Owen et al., 2016; Shim & Compton, 2020). The low self-report rate of mental illnesses could reflect several different aspects of inequitable dynamics, ranging from the racialized stigma associated with mental illness in racially and ethnically marginalized communities (Corrigan et al., 2003; Morrow et al., 2020), geographic and socio-economic access gaps to mental health care screening and treatment (Robles et al., 2019; Walker et al., 2016), and differential levels of comfort disclosing mental illness diagnoses at the time of incarceration (Corrigan et al., 2018; Reingle Gonzalez & Connell, 2014). This supported the notion presented by Sheehan et al., in saying the Black (Non-Hispanic) population did not seek professional help for mental illness (Sheehan et al., 2018). By getting help from friends, family, and church leaders, the necessity to seek medical interventions is minimized. The historical and ongoing abuse or mistreatment of the minority populations tend to direct a person in such group to seek community-based means of help. This mistrust can then lead to an underreporting of mental illness diagnoses and treatment in jails.

National jail healthcare accreditation organizations recommend mental health screenings for active suicidality (Berman & Canning, 2021; Folsom et al., 2006), or signs of schizophrenia (e.g., hallucinations) (Folsom et al., 2006), but there is less clarity in recommendations for screening for anxiety, depression, or trauma-related illnesses. When jails implement screening for mental illness, it is often part of a quality improvement, or implementation project, and even when successful, the experiences of jails are infrequently adopted by other jails (Gibbons et al., 2019; Juarros-Basterretxea et al., 2021; Proctor et al., 2021). The barriers in converting efficacious screening tools are likely multi-factorial, related to limited financial and administrative resources (Morris & Edwards, 2022), and de-prioritization of mental health due to competing health issues (Binswanger et al., 2009; Udo, 2019). The high prevalence of mental health conditions among inmates is overburdening a system, the criminal-legal system, which is not meant to be the primary avenue for mental health treatment.

The optimal timing of mental health screening during the jail admission process should be given a critical consideration. First, the process of being incarcerated is emotionally jarring, and feelings of depression and anxiety during the intake process may be reflective of an adjustment disorder rather than actual clinical depression, or anxiety. If initial screening results flag as concerning, individuals with criminal-legal involvement should be evaluated iteratively by clinicians with experience differentiating amongst these various psychiatric illnesses and presentations to ensure accurate diagnostic assessment and facilitate better guided treatment. Second, people who are incarcerated are often experiencing alcohol or drug withdrawal from substances that may be illicit, or prescribed. There is a clear solution to this issue—treatment of withdrawal symptoms and treatment of substance use disorder (Assistance TBoJ, 2022; Evans et al., 2022; Frank & Pollack, 2017). Notably, the Middlesex Jail & House of Correction implemented substance use disorder treatment with medications for opioid use disorder in October 2015 with extended release naltrexone, and in 2019 added other medications including methadone and buprenorphine (Evans et al., 2021; Ferguson et al., 2019; Stopka et al., 2022). Additionally, other jails in Massachusetts have published that implementation of substance use disorder in their jails decreased re-incarceration. (Evans et al., 2021, 2022; Matsumoto et al., 2022).

Individuals with criminal-legal involvement may be reluctant to report mental health symptoms or prior psychiatric treatments, including medications, for fear of negative consequences, like isolation. In addition, this could be the first mental health screening for some individuals who are undiagnosed with mental health illness. Historical and contemporary discrimination against Black individuals exacerbate distrust and mistrust of mental health care professionals (Saldana et al., 2021). Another factor that affects racially and ethnically minoritized populations is mental health access. A low percentage of mental health providers accept insurance, which has led to a larger divide in who has access to quality and timely psychiatric care (Cummings, 2015; Sloat, 2022). This was made more apparent with the onset of the COVID-19 pandemic, where several psychiatrists continue to use only telehealth, which marginalizing patients who do not have access to the required technologies (Sloat, 2022; Weiner, 2023). Even though improvements have been made on the diagnostic criteria for psychiatric disorders (bipolar disorder, major depression, and schizophrenia), there are racial and ethnic disparities, with minoritized populations more likely to receive incorrect diagnoses (Saldana et al., 2021). Compared to White individuals, racial/ethnic minorities receive lower quality care for their mental illness (Bailey et al., 2021; Sheehan et al., 2018). Due to experiences of racial discrimination in healthcare, many racial/ethnic minorities mute their ability to solicit for professional help (Bailey et al., 2021; Saldana et al., 2021; Sheehan et al., 2018). A nationally representative study (see Sheehan et al., 2018) found Black people may be less likely to seek mental health treatment both in the community and in jail. (Bailey et al., 2021; Sheehan et al., 2018; Snowden & Yamada, 2005) As a result of a long legacy of racism in the medical system, as well as a culturally stigmatized view of psychiatric illness and treatment in racially and ethnically minoritized populations, people who are Black (Non-Hispanic), Hispanic, Asian/Pacific Islander (Non-Hispanic), and American Indian may be less likely to take medications for mental illnesses.

We hope this report will encourage additional conversation about ways to improve mental health screening for ethnically and racially minoritized populations in jails sustainable and efficacious programs to connect minority community members to professional mental health resources. Investing to improve the identification and treatment of mental illnesses in jails can lead to improved life on re-entry into the community (Bonfine et al., 2020; Evans et al., 2022; Kennedy-Hendricks et al., 2016; Vinson et al., 2020). Linkage of people with mental health disorders to evidence-based treatment in jails and in the community is possible with adequate resource investment, community engagement, and by listening to the voices of people who experience incarceration and mental illness. The American Psychological Association (Stringer, 2019) and U.S. Department of Health and Human Services’ Substance Abuse and Mental Health Services Administration (Blandford & Osher, 2013) have published strategies focused on correctional settings improving the transition of mental health care of individuals with criminal-legal involvement from carceral settings to community. Peer navigation to assist linkage to care following release is one strategy (Hailemariam et al., 2020; Lincoln et al., 2006). A randomized controlled trial (see Kouyoumdjian et al., 2015) found that peer navigation helps to connect people to mental-health services on re-entry into the community (Barsky, 2022; Kouyoumdjian et al., 2015). Involving a racially and culturally diverse group of people with lived experiences of incarceration is key to any plan to address disparities in mental health care treatment for people leaving jail (McCracken, 2019; Reingle Gonzalez et al., 2019). Furthermore, including people with lived experience of incarceration on the research team when developing and implementing new treatment strategies should be the goal (Simon et al., 2021).

It is worth noting some research has reported linkage to mental health in the community is associated with increased risk of re-incarceration (Domino et al., 2019; Falconer et al., 2017). In these published works, authors describe the complex relationship between health care provision and the carceral system, as increased contact with mental health may lead to increased opportunities for “technical violations” (Domino et al., 2019). Technical violations are not new crimes, but are failures to comply with the “technical terms” of the release, such as abstaining from drugs, or verified consistent attendance of mental health appointments. Many violations come from not being compliant with conditions of mental health treatment. It is a self-defeating cycle of the parole process when missing a mental health appointment, often due to mental health crisis, lands an individual back in jail. People with mental illnesses are more likely to be re-incarcerated than people without mental illness because of a technical violation. (Eno Louden & Skeem, 2011). Further evaluations of mental health interventions focusing on post-release need to closely examine the outcomes and causes of re-incarceration.

Our study has limitations which deserve discussion. As the primary outcome is self-report of mental illness, the questions used are not part of any validated tool for identification of mental illness. It does, however, reflect on the person’s view of their mental health, which is an important perspective. Additionally, self-report may carry more weight as it is the perception of one’s well-being especially considering racially and ethnically minoritized populations are often untreated and left without access to formal mental health assessment (Saldana et al., 2021; Sheehan et al., 2018). Moreover, medications are not the only treatment for mental illnesses. There are several available treatments for mental illnesses, including, but not limited to, medications, psychotherapy, and neuromodulation therapy (i.e. ECT, TMS) (Martin et al., 2019). On the intake forms, individuals with criminal-legal involvement were not asked if they were receiving other types of treatment, like psychotherapy. As such, we may be underestimating the number of people who were engaged in mental health treatment.

Increased focus on systems and strategies aimed at interrupting the cycle of untreated mental illness and incarceration are necessary. In addition to decarceration and decriminalization of mental illness, evaluation of the current systems in place to address and treat mental illness during incarceration is crucial. Our research should serve as an additional siren, a warning of why the barriers to mental health evaluation in racially and ethnically minoritized communities highlight the perpetual nature of these inequities in access to care, and in further traumatization which directly impacts ongoing mental health.

### Supplementary Information

Below is the link to the electronic supplementary material.

Supplementary file1 (DOCX 18 KB)
