# Supplementary material for: Racial Differences in Self-Report of Mental Illness and Mental Illness Treatment in the Community: An Analysis of Jail Intake Data
Source: Adm Policy Ment Health. 2023 Sep 21;50(6):966–75. doi: 10.1007/s10488-023-01297-4 (PMC10543583; doi:10.1007/s10488-023-01297-4)
Supplement: Supplementary file 1 — Supplementary file1 (DOCX 18 KB) [file 10488_2023_1297_MOESM1_ESM.docx]

| **Table 1.** Bivariate and Multivariable Analyses of Reported Psychiatric Medications on Intake by Sociodemographic Characteristics (Internal note—this is everyone regardless of whether they had mental health treatment history) | | | | | | |
| --- | --- | --- | --- | --- | --- | --- |
|  | Cross-tabulations | | Bivariate regression analyses | | Multivariable regression analyses | |
|  | No (N = 6332)  N (%) | Yes (N = 1552)  N (%) | OR (95% CI) | P-value | AOR (95 % CI) | P-value |
| *Race* |  |  |  |  |  |  |
| White, Non-Hispanic | 3476 (75) | 1155 (25) | Reference |  | Reference |  |
| Black, Non-Hispanic | 1231 (89) | 154 (11) | 0.38 (0.31 – 0.45) | < 0.001 | 0.42 (0.35 – 0.51) | < 0.001 |
| Hispanic | 1430 (86) | 231 (14) | 0.49 (0.42 – 0.58) | < 0.001 | 0.69 (0.58 – 0.83) | < 0.001 |
| Asian/Pacific Islander  (Non-Hispanic) | 124 (95) | 7 (5) | 0.17 (0.08 – 0.36) | < 0.001 | 0.23 (0.11 – 0.50 | < 0.001 |
| Other race/ethnicity | 71 (95) | 5 (7) | 0.21 (0.09 – 0.53) | 0.001 | 0.31 (0.12 – 0.77) | 0.012 |
| *Age* |  |  |  |  |  |  |
| 18 – 29 years old | 1637 (87) | 244 (13) | Reference |  | Reference |  |
| 30 – 44 years old | 2845 (79) | 778 (21) | 1.83 (1.57 – 2.14) | < 0.001 | 1.72 (1.47 – 2.02) | < 0.001 |
| $\geq$45 years old | 1850 (78) | 530 (22) | 1.92 (1.63 – 2.27) | < 0.001 | 1.73 (1.45 – 2.05) | < 0.001 |
| *Foreign-Born*^a^ |  |  |  |  |  |  |
| No | 5179 (78) | 1423 (22) | Reference |  | Reference |  |
| Yes | 1153 (90) | 129 (10) | 0.27 (0.26 – 0.29) | < 0.001 | 0.52 (0.42 – 0.65) | < 0.001 |
| ^a^Outside of the 50 US States and Puerto Rico; *OR* Odds Ratio, *AOR* Adjusted Odds Ratio | | | | | |  |

**Supplemental Materials**
